# Severe Hemodynamic Instability in a Young Pregnant Woman with Massive Pericardial Effusion and Pulmonary Embolism Secondary to Primary Mediastinal Non-Hodgkin’s Lymphoma

**DOI:** 10.3390/jcm14082670

**Published:** 2025-04-14

**Authors:** Giuseppe Neri, Jessica Ielapi, Vincenzo Bosco, Helenia Mastrangelo, Federica Mellace, Nadia Salerno, Giuseppe Antonio Mazza, Giuseppe Filiberto Serraino, Daniele Caracciolo, Roberta Venturella, Daniele Torella, Pasquale Mastroroberto, Marco Chiappetta, Alessandro Russo, Pierosandro Tagliaferri, Pierfrancesco Tassone, Fulvio Zullo, Andrea Bruni, Federico Longhini, Eugenio Garofalo

**Affiliations:** 1 Department of Medical and Surgical Sciences, Magna Graecia University, 88100 Catanzaro, Italy; giuseppeneri91@gmail.com (G.N.); vincenzo.bosco@unicz.it (V.B.); heleniamastrangelo@gmail.com (H.M.); federica.mellace@gmail.com (F.M.); a.russo@unicz.it (A.R.); flonghini@unicz.it (F.L.); eugenio.garofalo@unicz.it (E.G.); 2 Department of Experimental and Clinical Medicine, Magna Graecia University, 88100 Catanzaro, Italy; jessica.ielapi22@gmail.com (J.I.); nadia.salerno@unicz.it (N.S.); giuseppeantoniom@gmail.com (G.A.M.); serraino@unicz.it (G.F.S.); d.caracciolo@unicz.it (D.C.); venturella@unicz.it (R.V.); dtorella@unicz.it (D.T.); mastroroberto@unicz.it (P.M.); tagliaferri@unicz.it (P.T.); tassone@unicz.it (P.T.); zullo@unicz.it (F.Z.); 3 Department of Health Sciences, Magna Graecia University, 88100 Catanzaro, Italy; marco.chiappetta@unicz.it

**Keywords:** mediastinal syndrome, ECMO, pulmonary embolism, non-Hodgkin lymphoma, pregnancy, cardiac tamponade, anticoagulation

## Abstract

**Background:** Lymphomas account for approximately 10% of cancers diagnosed during pregnancy, with Hodgkin’s lymphoma being the most common. However, non-Hodgkin lymphomas, including primary mediastinal large B-cell lymphoma (PMBCL), also represent a significant proportion. Both mediastinal lymphomas and pregnancy develop a hypercoagulable state, increasing the risk of venous thromboembolism and massive pulmonary embolism (PE), requiring extracorporeal membrane oxygenation (ECMO). **Methods:** Clinical data, blood test and imagings have been collected by the medical records of the patient. **Results:** We present a 25-year-old woman, at 32 weeks of gestation, who presented to the emergency department with progressive dyspnea and asthenia. Echocardiography revealed a hemodynamically significant pericardial effusion and severe right ventricular dysfunction. Given the severity of her condition, she underwent an emergency caesarean section and subsequently a pericardial drainage. A chest computed tomography scan revealed an incidental mediastinal mass along with a massive PE. Despite pericardial drainage, she remained hemodynamically unstable. Since thrombolysis was contraindicated for the recent cesarean section, venoarterial ECMO was initiated. Systemic anticoagulation was guaranteed by heparin, which shifted to argatroban for heparin resistance. The mediastinal mass was also biopsied, and the diagnosis of PMBCL carried out. Cytoreductive chemotherapy was initiated with the COMP-R regimen (i.e., cyclophosphamide, vincristine, methotrexate, prednisone, and rituximab), and the patient progressively improved up to ICU and hospital discharge. **Conclusions:** This case highlights the challenges in managing a complicated patient requiring early multidisciplinary intervention, which was crucial for stabilizing the patient and optimizing fetal and maternal prognosis.

## 1. Introduction

Lymphomas represent approximately 10% of cancers diagnosed during pregnancy, with Hodgkin’s lymphoma being the most common. However, aggressive non-Hodgkin lymphomas (NHLs), including primary mediastinal large B-cell lymphoma (PMBCL), account for a significant proportion [1]. The incidence of NHLs in pregnancy is estimated to be 1 in 6000 pregnancies, with PMBCL comprising 7–12% of diffuse large B-cell lymphoma (DLBCL) cases [2]. The increasing maternal age at first pregnancy may contribute to a higher occurrence of pregnancy-associated lymphomas [3].

Mediastinal lymphomas frequently present with superior vena cava syndrome, dyspnea, orthopnea, cough, or chest pain [4]. Pregnancy itself is a prothrombotic state [5], and when combined with lymphoma-associated hypercoagulability, the risk of venous thromboembolism (VTE) is significantly heightened [6]. Pulmonary embolism (PE) remains a leading cause of maternal mortality in oncologic pregnancies. According to ESC guidelines [7], venoarterial extra-corporeal membrane oxygenation (va-ECMO) is recommended in cases of massive PE with hemodynamic instability when thrombolysis or catheter-directed therapy is contraindicated or ineffective. We present and discuss the case of a young pregnant patient who developed cardiogenic shock due to cardiac tamponade and massive PE. She was managed with a multidisciplinary approach, including va-ECMO support, and subsequently underwent cytoreductive therapy following a new diagnosis of PMBCL, with a favorable clinical outcome.

## 2. Case Presentation

A 25-year-old woman at 32 weeks of gestation (70 kg, 165 cm), with a history of pericarditis six months earlier, presented to the emergency department with dyspnea and asthenia. Echocardiography revealed a hemodynamically significant pericardial effusion with signs of tamponade. She was immediately intubated and underwent an emergency caesarean section due to progressive clinical deterioration. Thereafter, the cardiac surgeon performed a pericardiocentesis with the drainage of 400 mL of serosanguinous fluid with the insertion of a pericardial tube. The patient remained hemodynamically unstable (MAP < 65 mmHg). A total-body CT scan revealed the presence of massive PE with emboli in the right main pulmonary artery and left secondary branches, a 20 × 10 cm mediastinal mass compressing adjacent structures, and left internal jugular vein thrombosis. Due to persistent hemodynamic instability refractory to inotropic and vasopressor support, va-ECMO was initiated (CardioHelp, Getinge, Solna, Sweden), with cannulation via the right femoral vein (drainage) and left femoral artery (reinfusion), maintaining an ECMO blood flow > 3.5 L/min [7]. A distal perfusion catheter was also placed under ultrasound guidance into the superficial left femoral artery with a short 6 Fr armored cannula [8]. Initial anticoagulation therapy consisted of a continuous heparin infusion, which was a few hours later switched to argatroban (i.e., a direct thrombin inhibitor) at 2 mcg*kg/min due to high thrombotic risk [9]. Argatroban was titrated to maintain an activated partial thromboplastin time (aPTT) ranging between 1.5 and 2 times the baseline value [9]. Cardiac ultrasonography was also performed, showing persistently elevated pulmonary pressures, thus prompting the initiation of (4–20 ppm) inhaled nitric oxide therapy [10,11]. Given the increased risk of postpartum hemorrhage, thrombolysis was not performed [12]. Cerebral oximetry (ForeSight, Edwards Lifesciences Corporation, Irvine, CA, USA) was closely monitored to detect asymmetrical or bilateral drop in cerebral saturation to recognize any neurological complications for Harlequin syndrome [13,14,15]. Protective lung ventilation and hemodynamic support with tartrate norepinephrine (0.5 mcg/kg/min) and dobutamine (3 mcg/kg/min) were administered [7,16].

Since the diagnosis of the mediastinal mass was new, the patient was transferred to the operating room on Day 1 for a mediastinal mass biopsy during va-ECMO treatment. The histopathological findings diagnosed the presence of a PMBCL (Day 7).

On Day 2, pulmonary angiography excluded the presence of macroscopic evidence of thrombi in the left lung, while an endoluminal thrombus was present in the upper and middle lobal branches of the right pulmonary artery, without limitation of the blood flow.

Following hemodynamic improvement, on Day 3, the patient was weaned off va-ECMO [8]. The criteria for va-ECMO weaning are based on those outlined by the Extracorporeal Life Support Organization (ELSO). Specifically, we considered va-ECMO weaning when patients demonstrated sufficient cardiac recovery, requiring only minimal vasoactive and inotropic support to maintain an adequate pulse pressure (>10 mm Hg) and a mean arterial pressure (>65 mm Hg) [8]. During the entire course of ICU care, the patient was also closely monitored with bedside imaging techniques, such as lung ultrasonography and electrical impedance tomography, beyond the chest X-ray [17,18,19].

On Day 8, after communication of the histopathological finding from the mass biopsy (i.e., a PMBCL), the oncologists provided an indication for cytoreductive therapy (COMP-R regimen: cyclophosphamide, vincristine, methotrexate, prednisone, and rituximab) that was started on Day 9 [20]. In addition, due to the unavailability of argatroban, anticoagulation was switched to the continuous infusion of bivalirudin at 0.02 mg*kg/h [21].

On Day 13, the oxygenation indices slightly deteriorated, with the new onset of pulmonary infiltrates in the lung ultrasonography and modifications of the ventilation distribution in the electrical impedance tomography [17,18,19]. Flexible bronchoscopy with broncho-alveolar lavage was performed; the specimen was analyzed with the film array technique and Acinetobacter baumanii was identified (later also confirmed by standard culture). Therefore, antimicrobial therapy with cefiderocol was initiated [22].

Due to the time spent under invasive mechanical ventilation and the expectation of a prolonged weaning, on Day 15, the patient was tracheostomized with a modified Ciaglia technique. On Day 18, the cardiac ultrasonographic assessment revealed the presence of a thin circumferential pericardial effusion without hemodynamic compromise. The right atrium and ventricle were at the upper limit of the normal range of dimensions, with preserved systolic function. The persistence of fever and neutropenia imposed the initiation of a prophylactic antimicrobial therapy with ganciclovir, trimethoprim-sulfamethoxazole, and fluconazole. Oncologists recommended the administration of granulocyte colony-stimulating factor until the neutrophil count was >1000/mm^3^ [23].

On Day 24, the patient underwent a second chemotherapy cycle. The day after, she was set in spontaneous breathing with a high flow, humidified, and heated air–oxygen admixture through tracheostomy. On Day 30, the patient was transferred to the oncology ward for further treatment and then discharged alive at home without tracheostomy.

**Figure 1:**
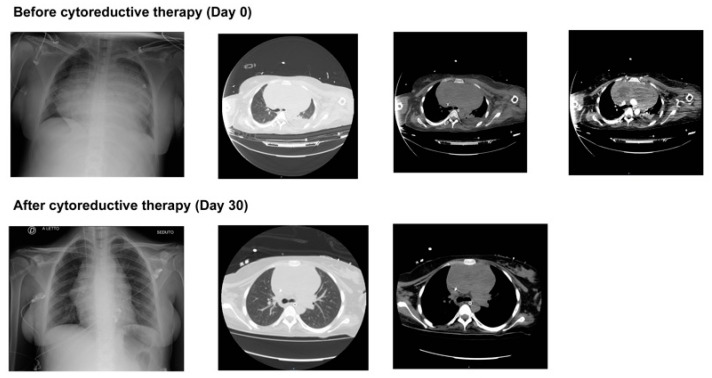
Chest imaging at ICU admission and after cytoreductive therapy demonstrates the evolution of both acute lung injury and the reduction of the mediastinal mass.

The chest imaging at ICU admission and discharge are shown in Figure 1, showing the reduction in the dimensions of the mediastinal mass. Blood tests of main interest through hospital admission are also shown in Table 1.

Written informed consent for the publication of individual anonymous data was obtained by the patient.

## 3. Discussion

We present the case of a young pregnant woman who required a rapid and complex multidisciplinary approach involving intensivists, gynecologists, cardiac and thoracic surgeons, infectious disease specialists, and oncologists. This collaboration was essential in managing severe hemodynamic instability caused by pericardial effusion with tamponade and massive PE, ultimately linked to a newly diagnosed PMBCL. The coordinated efforts of multiple specialists enabled the timely exchange of expertise and well-informed clinical decisions, which were critical to navigating such a complex clinical scenario.

The clinical management of this case was challenging. First, the patient was pregnant. When she presented at the Emergency Department, she showed asthenia and progressive dyspnea, symptoms that could be attributed to common pregnancy-related conditions such as anemia or physiologic cardiopulmonary adaptations [24]. However, the concomitant hemodynamic instability led to the recognition of cardiac tamponade and right ventricular dysfunction, necessitating a rapid intervention. Since the pericardiocentesis may be complicated by events requiring major cardiac surgery [25], after discussion with the cardiac surgeons, the gynecologists gave indication to an emergent cesarean section, followed by the pericardiocentesis. This was mainly dictated by the need to first reduce the time to its minimum for the fetus placental hypoperfusion, consequent possible hypoxic damage, and other complications that acutely threaten the life of both fetus and/or mother [26]. In fact, the American College of Obstetricians and Gynecologists (ACOG) committee on professional standards and the National Institute of Clinical Excellence (NICE) guidelines suggest that decision-to-delivery interval and emergency cesarean section should not exceed 30 min to reduce maternal or fetal compromise and poor outcome [27]. It should be noted that the fetus was 32 weeks, a preterm infant with increased risk for respiratory distress syndrome at birth, increased morbidity, and mortality [28]. Given that there was not enough time to administer antenatal steroid prophylaxis [29], an exogenous surfactant would have been administered [30]. The preterm newborn did not require any intensive treatment other than the surfactant administration and continuous positive airway pressure for a few hours after surfactant administration; the newborn was discharged from the Neonatal Intensive Care Unit after 12 days, with outcomes resembling those reported in a larger similar population of patients [31].

Soon after the cesarean section, the cardiac surgeon conducted a pericardiocentesis with indwelling pericardial drainage. It was expected that the hemodynamic instability improved, but this occurred only partially. Persistent hypotension after pericardiocentesis should raise suspicion for cardiac perforation, coronary laceration, vasovagal response, and finally pericardial decompression syndrome or stress cardiomyopathy, two under-recognized diagnoses [32]. Based on further emergent transthoracic echocardiography imaging and blood gas analysis, it was mandatory to look for other reasons of hypotension and respiratory distress with a CT scan, a diagnostic approach that at that time was possible since the preterm was born. Untreated massive PE with hemodynamic instability is a life-threatening emergency, with maternal mortality exceeding 20% [33]. Although systemic thrombolysis is the first-line treatment, in our case, it was contraindicated due to the high risk of hemorrhage following emergency cesarean section [34]. Furthermore, in cases of massive pulmonary embolism (PE) with shock, the failure of systemic thrombolysis or contraindications to it, catheter-based or surgical embolectomy should be considered [35]. Given that our center serves as the regional ECMO referral center, and considering the patient’s severe hemodynamic instability and hypoxemia, we opted to initiate extracorporeal life support [36,37]. For this reason, we decided to run va-ECMO to maintain organ perfusion and to allow for hemodynamic stabilization while minimizing the need for high-dose vasopressors. In addition, va-ECMO significantly improves survival in massive PE with cardiogenic shock when thrombolysis cannot be performed [36,37].

Anticoagulation management was also crucial due to the fine balance between bleeding risk for the recent cesarean section and prothrombotic status related to the pregnancy and the presence of the mediastinal mass [38]. At the beginning, continuous unfractionated heparin infusion was started; however, due to the difficulty of reaching the current range of anticoagulation (i.e., APTT between 1.5 and 2 times the baseline value) despite a normal value of antithrombin-III, we decided to shift to argatroban, which allows for a precise titration of the anticoagulation. Although unfractionated heparin is the most widely used anticoagulant during ECMO [39], argatroban can be used as an alternative anticoagulant such as in patients with heparin-induced thrombocytopenia or heparin resistance [40,41,42]. In the recent past, argatroban has even been used in a 17-week pregnant woman with acute respiratory distress syndrome for severe tuberculosis [43], and in patients with cardiogenic shock with concomitant deep vein thrombosis and PE [44]. It should be argued that we used argatroban rather than other anticoagulants such as bivalirudin [21]. Bivalirudin has a half-life of approximately 25 min, whereas argatroban has a half-life of about 45 min. While this shorter half-life might seem advantageous for bivalirudin due to its greater manageability, it is not necessarily beneficial in va-ECMO settings. The relatively short half-life of bivalirudin can lead to decreased drug concentrations in areas of blood stagnation, such as poorly contracting cardiac chambers during va-ECMO, potentially increasing the risk of clot formation [45].

The incidental discovery of a mediastinal mass that led to the diagnosis of PMBCL was the last challenge we had to face, even though this was the real etiology of all comorbidities. PMBCL is a rare but aggressive non-Hodgkin lymphoma with an incidence of 1 in 6000 pregnancies [2], which in our patient caused superior vena cava syndrome, respiratory distress, and increased thromboembolic risk due to vascular compression and inflammation [4]. The combination of pregnancy-related hypercoagulability and lymphoma-induced prothrombotic state significantly increased the risk of PE, which remains a leading cause of maternal mortality in oncologic pregnancies [46]. In addition, the timely initiation of treatment was crucial [47]. In our case, we had to start the chemotherapeutic treatment even if the patient was intubated, since it was not possible to proceed with weaning and extubation due to mass compression of the airways. In accordance with the current recommendations for aggressive lymphomas in pregnancy, the oncologist opted for the COMP-R regimen (cyclophosphamide, vincristine, methotrexate, prednisone, and rituximab) [20]. As demonstrated in Figure 1, the mediastinal mass was largely reduced, the patient could be weaned off the mechanical ventilator, and the percutaneous tracheostomy was removed.

In the literature, only a few cases of PMBCL during pregnancy have been reported. Some involved patients in the first or second trimester [48,49], who received a CHOP-like regimen (i.e., cyclophosphamide–hydroxydaunorubicin–oncovin–prednisone) during pregnancy. Another case, presenting with concomitant superior vena cava obstruction, was diagnosed in the third trimester, and chemotherapy with a CHOP-like regimen was administered following a cesarean section [50]. Additionally, a nulliparous woman at 26 weeks of gestation was reported with PMBCL and inferior vena cava obstruction, accompanied by hemodynamic instability managed with pharmacological support [51]. Although all of these cases involved multidisciplinary teams, our patient also required the involvement of cardiac surgeons and the ECMO team due to severe hemodynamic instability and hypoxemia—a scenario that, to the best of our knowledge, has not been previously reported.

To effectively address the needs of critically ill patients, a multidisciplinary team including physicians and nurses from various specialties, plays a crucial role in providing personalized care [52]. In the case presented, we adopted a comprehensive approach that integrated expertise from intensivists, gynecologists, cardiac and thoracic surgeons, infectious disease specialists, and oncologists. Encouraging collaboration among different specialists fosters the exchange of knowledge and experience, which is essential for making prompt and well-balanced clinical decisions, particularly in complex medical situations like this one.

## Figures and Tables

**Table 1:** Blood test during the acute phase in ICU.

Parameter	Normal Range	Day 0	Day 7	Day 14	Day 21	Day 28
WBC (×10^3^/mL)	5.2–12.4	20.47	13.84	5.28	7.01	2.45
Neutrophils (%)	40–74%	89.9	85.5	91.1	76.1	71.4
Lymphocytes (%)	19–48	3.3	5.6	4.1	9.7	21
RBC (×10^6^/μL)	4.2–5.9	4.15	2.93	2.94	2.73	3.41
Platelets (*n* × 10^3^/μL)	130–400	236	205	315	338	454
Procalcitonin (ng/mL)	<0.5	1.54	0.06	0.74	0.23	0.22
Creatinine (mg/dL)	0.81	0.35	0.18	0.24	0.15	0.21
ALT (UI/L)	4–36	368	26	39	16	16
Total Bilirubin (mg/dL)	0.1–1.2	0.36	0.50	0.30	0.19	0.26
aPTT (seconds)	22.0–36.6	28	44	31	31	31

WBC, white blood cells; RBC, red blood cells; ALT, alanine aminotransferase; aPTT, activated partial thromboplastin time.

## Data Availability

The original contributions presented in this study are included in the article. Further inquiries can be directed to the corresponding author.
